# Accessory Mental Foramen in a Patient with Mandibular Bisphosphonate-Related Osteonecrosis of the Jaw (BRONJ) Lesion: A Case Report

**DOI:** 10.29252/wjps.9.1.92

**Published:** 2020-01

**Authors:** Sahand Samieirad, Elahe Tohidi

**Affiliations:** 1 Oral and Maxillofacial Diseases Research Center, Mashhad University of Medical Sciences, Mashhad, Iran; 2 Oral and Maxillofacial Surgery Department, Mashhad Dental School, Mashhad University of Medical Sciences, Mashhad, Iran; 3 Oral and Maxillofacial Radiology Department, Oral and Maxillofacial Diseases Research Center, Mashhad University of Medical Sciences, Mashhad, Iran

**Keywords:** Accessory mental foramen, Paresthesia, Mandible, Bisphosphonate, Osteonecrosis, Jaw

## Abstract

The mental nerve is a sensory nerve which traverses through mental foramen to innervate the lower lip, chin skin and the mandibular labial gingiva. Interestingly, it’s variant such as the accessory mental foramen (AMF) was described as an unusual finding in the recent literature. Hereby, we reported a patient who was operated to treat the mandibular bisphosphonate-related osteonecrosis of the jaw (BRONJ) lesion. Intraoperatively, an accessory mental foramen was detected posterior to the main foramen and nerve, on the right side of the mandible. This case report highlighted the necessity for proper radiological and clinical evaluation of mental foramina in order to avoid nerve injury and postoperative paresthesia. The review of the literature and the clinical findings were also discussed in this article.

## INTRODUCTION

The mental foramen (MF) is a significant anatomical landmark on the anterolateral surface of the mandible near the chin.^1^^,^^2^ Normally, the location of MF is near the premolars apices.^3^^-^^5^ In anesthetic techniques such as the incisive/mental nerve block, the abovementioned location of MF plays the role of a reference.^2^^,^^6^^,^^7^ The mental nerve supplies sensation to the lower lip, mandibular canines and premolars as well as the labial mucosa and the skin of chin area.^6^^-^^8^ The recent articles introduced the accessory mental foramen as an anatomical variation which is rare and unusual.^3^^,^^6^^,^^9^^,^^10^ Considering the surgical and clinical importance of the accessory mental foramen and nerve, we decided to report an interesting case of AMF which was intra-operatively detected in an old male patient who underwent an operation for bisphosphonate-related osteonecrosis of the jaw (BRONJ) lesion on the right side of the mandible. 

## CASE REPORT

**Fig. 1A, B:**
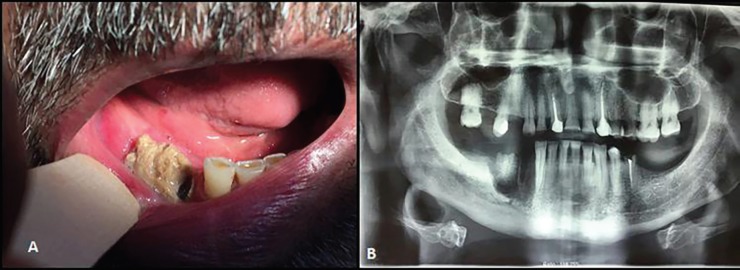
Preoperative clinical and panoramic view of BRONJ

A 56-year-old male patient was referred to the Department of Oral and Maxillofacial Surgery of Mashhad Dental School, Mashhad, Iran in January 2017. The patient’s chief complaint was pain and infection of exposed bone on the right posterior mandible since 2 months ago (Figure 1A). The panoramic radiography illustrated the necrotic bone segment within the ill-defined radiolucency in the above-mentioned region (Figure 1B). Regarding the past medical history, the patient had suffered multiple myeloma in his lumbar spines and pelvis. He was treated with intravenous bortezomib (1.25 mg/m2, days 1, 4, 7, 11) and intravenous zolenderonate bisphosphonate drug (4 mg/monthly, for 12 months). 

The patient had no history of the steroid therapy, malnutrition and the radiotherapy to the craniofacial region. Therefore, the probability of osteomyelitis, osteoradionecrosis, and steroid-induced necrosis was ruled out. Unfortunately, in the past dental history, the patient had painful right lower premolars which were extracted (teeth 44 and 45) by a general dentist without bisphosphonate drug holiday and discontinuation. Considering the clinical and radiographic examinations as well as past medical and dental history, the diagnosis of bisphosphonate-related osteonecrosis of the jaw (BRONJ) was confirmed. Moreover, the bacterial culture test results of the region were negative.

**Fig. 2:**
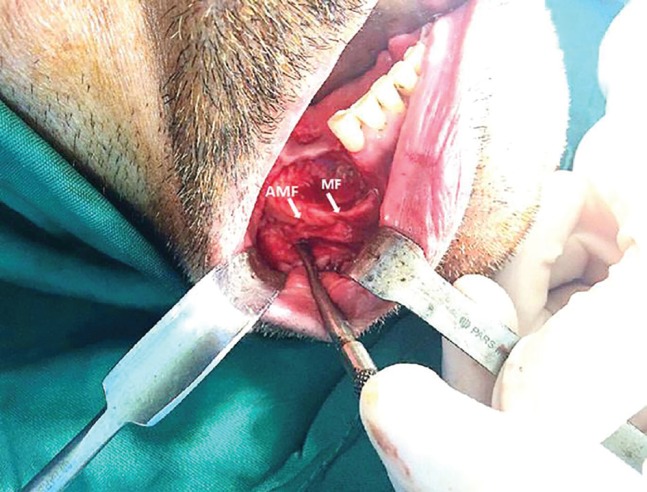
The accidental diagnosis of AMF posterior to main mental foramen intraoperatively

After an accurate evaluation, the present lesion was determined to be in the stage 2 of BRONJ (stage 2 was distinguished by painful areas of exposed bone accompanied by soft tissue or bone inflammation or infection). The informed consent was obtained from the patient. Then, upon consultation with the internist, the antibiotic therapy with penicillin and analgesic therapy with acetaminophen were advised for the patient. In addition, the zolenderonate drug was discontinued for 3 months. The protocol was reviewed and approved by our Institutional Review Board. Thus, the surgeon decided to perform the debridement and sequestrectomy of the mandibular lesion under general anesthesia. During the operation and after the flap elevation, the surgeon accidentally observed the accessory mental foramen (AMF) with an additional nerve, right posterior to the main MF (Figure 2).

It should be noted that this landmark was not detected by the panoramic view preoperatively. Both nerves (main and accessory mental nerves) were preserved intra-operatively, without any iatrogenic injury or sensory complication. The debridement of the lesion was thoroughly performed and the buccal flap was subperiosteally dissected and undermined. Afterwards, the flap was sutured without any tension to achieve the precise passive soft tissue coverage. No histological evaluation was conducted on the removed bone portions from the mandible in this case as the diagnosis of BRONJ was previously confirmed by clinical examination.

**Fig. 3:**
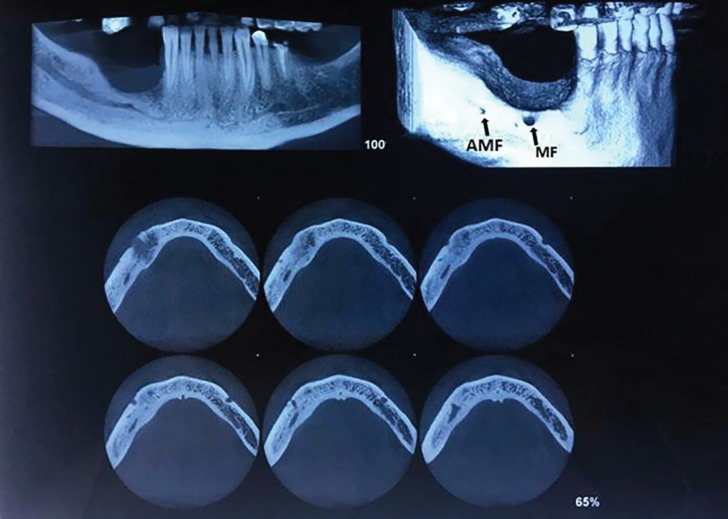
The diagnosis of AMF posterior to main mental foramen confirmed by CBCT

**Fig. 4:**
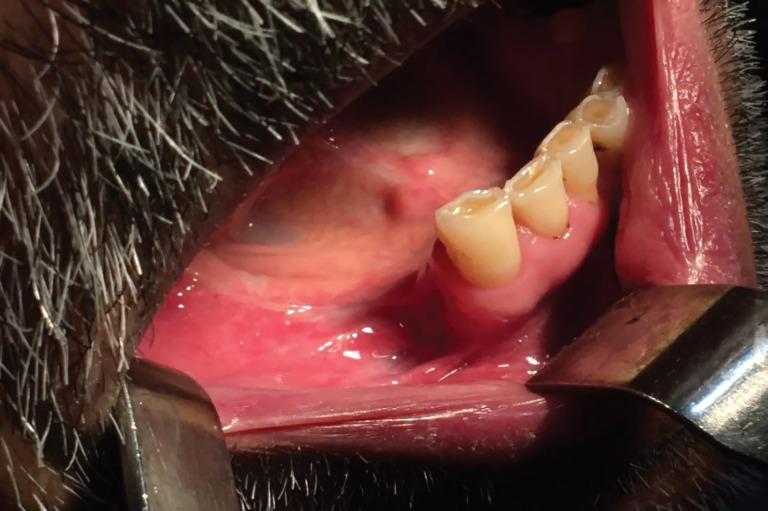
The postoperative view of patient with satisfying repair of the lesion

The healing of the lesion was uneventful, one month postoperatively. The CBCT (Cone Beam Ct-Scan) was requested postoperatively for further study of AMF. Using the CBCT views, the accessory mental foramen was obviously detected just posterior to the main one on the right side of mandible (Figure 3). The one-year follow-up revealed no postoperative complication. Neither the flap dehiscence nor the mental nerve paresthesia was observed in the operated area (Figure 4)

## DISCUSSION

The mental nerve divides into four sensory branches as follows: Medial and lateral inferior labial branches which innervate the lower lip skin, oral mucosa and gingiva as far posterior as the second premolar, angular and mental branches which innervate the angle of the mouth and the skin of the mental region, respectively.^6^^,^^9^^,^^11^ The ability to differentiate an accessory mental foramen from a nutrient one is a crucial attribute for clinicians.^12^^,^^13^ The origin of nutrient foramen is not the mandibular canal and also its dimensions are relatively smaller.^3^^,^^6^

According to Naitoh and Katakami *et al.*, an accessory foramen connecting, to various extents, with the mandibular canal is an accessory mental foramen (AMF).^12^^,^^13^ On the contrary, an accessory foramen having no sort of connection with the mandibular canal is a nutrient foramen.^12^^,^^13^ The incidence of AMF presence varies within the range of 1.5 % to 14.3% in regard to the recent articles.^1^^,^^3^^,^^5^^,^^9^^,^^11^^,^^14^^-^^16^ It should be noted that the patient’s age and gender as well as the mandibular side were not documented to have significant statistical relations with AMF incidence.^2^^,^^9^

Double and triple MFs were reported in 1.8-10.6% and 0.6-1.2% of Brazilian population. Furthermore, the bilateral accessory mental foramina were seen in 1.1% of these people.^17^ Gershenson *et al.* studied 525 dry mandibles of Israeli population. Their reports indicated that 4.3% of the mandibles had a double MF and 0.7% had triple MF.^18^ Voljevica *et al.* asserted that the incidence rate of AMF was 2.7% in Bosnia and Herzegovina population by Observing 150 adult dry human mandibles.^19^ Singh and Srivastav reported the incidence of AMF to be 7.2% to 13% in dry adult Indian human mandibles.^20^

Kalender *et al.* reported the presence of AMF in 6.5% of the Turkish population.^21^ Zmysłowska-Polakowska *et al.* examined mandibular CBCTs of 200 Polish patients (400 sides at all). These authors reported that the incidence rate of AMF was 7%.^2^ Following the identification of an accessory mental foramen, more attention should be paid to conservative and gentle surgical manipulation in order to avoid the neurosensory disturbances.^1^^,^^2^^,^^6^ Singh and Srivastav reported that the accessory mental foramina were found on the left sides of dry human mandibles in 8% of the cases and in 5 % of the subjects on the right side.^20^ On the other hand, Voljevica *et al.* stated that AMF was only observed on the right side of the mandible.^19^ Also AMF occurrence has been reported to be 3.52% of and 4.22% on the left and right side of the mandible respectively in accordance with a study performed on 142 South Indian mandibles by Roopa *et al.*^10^ These reports^10^^,^^19^ were in agreement with our study finding. The review of important and interesting case reports about the AMF observation during the oral and maxillofacial or implant surgeries were listed in Table 1.^1^^,^^4^^,^^6^^,^^14^^-^^16^^,^^22^^-^^26^ In fact, none of them reported AMF in a BRONJ case (Table 1). 

**Table 1:** The review of important and interesting case reports about the AMF observation during the oral and maxillofacial or implant surgeries

**No**	**Author** **(Year)**	**Patient’s nationality**	**Number of reported AMF cases**	**Patient’s age** **(Gender)**	**No. of mental foramina**	**Location of AMF**	**AMF Diagnosis** **time**	**Type of maxillofacial surgery **
1	Jha *et al.* (2012)	Indian	1	75 (F)	2	Right side of the mandible	Intra- operatively	Peripheral neurectomy of the mental nerve
2	Mamatha *et al.* (2013)	Indian	1	22 (M)	3	Left side of the mandible	Intra-operatively	ORIF
3	Garay *et al.* (2013)	American	2	44 (F)/60 (M)	2/2-2	Right side of the mandible/ Bilaterally	Pre operatively	Implant surgery
4	Sekerci *et al.* (2014)	Turkish	1	42 (M)	3/2	Bilaterally	Pre operatively	Implant surgery
5	Mihaylova (2014)	Bulgarian	1	33 (F)	3	Right side of the mandible	Pre operatively	Implant surgery
6	Aykol *et al.* (2015)	Turkish	1	44 (F)	2	Left side of the mandible	Pre operatively	Implant surgery
87	Torres *et al.* (2015)	Brazilian	1	63 (M)	2	Right side of the mandible	Pre operatively	Implant surgery
9	Gopinath *et al.* (2015)	Indian	1	23 (M)	2	Right side of the mandible	Intra-operatively	ORIF
10	Sahoo *et al.* (2016)	Indian	1	20 (M)	2	Left side of the mandible	Intra-operatively	ORIF
11	Ravi *et al.* (2017)	Indian	1	20 (M)	2	Left side of the mandible	Intra-operatively	ORIF
12	Bhata (2017)	Indian	1	43 (F)	2/2	Bilaterally	Pre operatively	Surgical removal of the impacted tooth and root
13	Goyushov *et al.* (2017)	Turkish	1	61 (M)	3/3	Bilaterally	Pre operatively	Implant surgery
14	Kabir *et al.* (2017)	Bangladeshi	1	64 (M)	2	Right side of the mandible	Pre operatively	Not reported

M: Male, F: Female, AMF: accessory mental foramen, ORIF: open reduction and internal fixation

The present study was consistent with most papers which reported that the AMF was observed on the right side of mandible right posterior to the main foramen.^1^^,^^3^^,^^5^^,^^9^^,^^11^^,^^14^^-^^16^ The presence of the accessory mental nerve can cause some surgical difficulties and neurosensory complications during different oral and maxillofacial surgeries such as dento-alveolar and implant surgeries, open reductions of mandibular fractures, genioplasty and segmental orthognathic surgeries.^3^^,^^11^^,^^12^^,^^15^ Every Multiple myeloma case must first be treated with the standard anti-tumor therapy. Bisphosphonates are one of the pharmacological agents currently recommended for its treatment and prevention.

Multiple myeloma is a systemic malignancy of plasma cells that typically involves the multiple sites of body within the bone marrow. It is a type of bone marrow cancer affecting several areas such as the spines, skull, pelvis and ribs.^27^^,^^28^ The multiple myeloma lesions must be initially treated with the standard anti-tumor or proteasome inhibitor drugs according to recent protocols.^27^^,^^28^ Although bisphosphonates (BP) are not the first line therapy in multiple myeloma.^27^ they are used in combination with other drugs if a myeloma bone disease exists.^27^^,^^28^ (1, 2). Thus, the intravenous bortezomib (proteasome inhibitor drug) with zolenderonate (BP) were administered by the internist for this case.

The mechanism by which the bisphosphonates inhibit osteoclastic bone resorption is different from that of other anti-resorptive agents. Bisphosphonates attach to hydroxyapatite binding sites on bony surfaces, especially surfaces undergoing active resorption.^29^ They can pose a danger to the ability of the osteoclasts to form the ruffled borders and can prevent them from adhering to the bony surface for bone resorption.^30^^,^^31^ Bisphosphonates also decrease the osteoclast activity by declining the osteoclast progenitor development and recruitment and by assisting the osteoclast apoptosis.^30^^,^^31^ In addition, bisphosphonates appear to have a beneficial effect on osteoblasts.^29^

It is worth noting that the high dose or long-term use of BP, especially the IV forms, might result in adverse effects such as bisphosphonate-related osteonecrosis of the jaw (BRONJ). This complication was reported by Marx for the first time in 2003.^31^ According to the papers, the inferior alveolar nerve can be involved in early stages of only limited BRONJ types.^29^ However there is no correlation between the branching of the inferior alveolar nerve or mental nerve and BRONJ occurrence,^29^ which is in agreement with our case. This case report highlighted the necessity for careful preoperative radiographic assessment of the mandibular canal and foramen to find out any accessory nerves. 

To sum up, it can be concluded that the awareness of oral and maxillofacial surgeons about the regular anatomy and radiographic identification of the MF anatomic variations is a highly essential issue and must be highlighted. Moreover, it is important to avoid the injury to neurovascular bundles passing through AMF intra-operatively as this iatrogenic damage might lead to disturbing postoperative paresthesia or dysesthesia.
